# Identification and functional analysis of SOX10 phosphorylation sites in melanoma

**DOI:** 10.1371/journal.pone.0190834

**Published:** 2018-01-09

**Authors:** Julia C. Cronin, Stacie K. Loftus, Laura L. Baxter, Steve Swatkoski, Marjan Gucek, William J. Pavan

**Affiliations:** 1 Genetic Disease Research Branch, National Human Genome Research Institute, National Institutes of Health, Bethesda, MD, United States of America; 2 Proteomics Core, National Heart, Lung, and Blood Institute, National Institutes of Health, Bethesda, MD, United States of America; Rutgers University, UNITED STATES

## Abstract

The transcription factor SOX10 plays an important role in vertebrate neural crest development, including the establishment and maintenance of the melanocyte lineage. SOX10 is also highly expressed in melanoma tumors, and SOX10 expression increases with tumor progression. The suppression of SOX10 in melanoma cells activates TGF-β signaling and can promote resistance to BRAF and MEK inhibitors. Since resistance to BRAF/MEK inhibitors is seen in the majority of melanoma patients, there is an immediate need to assess the underlying biology that mediates resistance and to identify new targets for combinatorial therapeutic approaches. Previously, we demonstrated that SOX10 protein is required for tumor initiation, maintenance and survival. Here, we present data that support phosphorylation as a mechanism employed by melanoma cells to tightly regulate SOX10 expression. Mass spectrometry identified eight phosphorylation sites contained within SOX10, three of which (S24, S45 and T240) were selected for further analysis based on their location within predicted MAPK/CDK binding motifs. SOX10 mutations were generated at these phosphorylation sites to assess their impact on SOX10 protein function in melanoma cells, including transcriptional activation on target promoters, subcellular localization, and stability. These data further our understanding of SOX10 protein regulation and provide critical information for identification of molecular pathways that modulate SOX10 protein levels in melanoma, with the ultimate goal of discovering novel targets for more effective combinatorial therapeutic approaches for melanoma patients.

## Introduction

SOX10 (SRY-box 10) is a multipotent transcription factor required for survival, proliferation and differentiation of a wide variety of cells, including neural crest-derived melanocytes, peripheral nervous system neurons and glia, and oligodendrocytes of the central nervous system. Individuals with *SOX10* mutations present clinically with the neurocristopathies Waardenburg syndrome (WS) 4C, WS2E, and PCWH (peripheral demyelinating neuropathy, central demyelination, WS, and Hirschprung disease) [1–8]. In addition, SOX10 is highly expressed in melanoma tumors, is rarely mutated in melanoma, and SOX10 knockdown in melanoma cells and tumors causes interrupted cellular proliferation, growth arrest, and reduced tumor size *in vivo* [9–11]. Thus maintenance of SOX10 expression is important in tumor initiation, maintenance, and progression to advanced stages of melanoma. SOX10 protein is also highly expressed in breast, glioma, glioblastoma multiforme, salivary adenoid cystic tumors and hepatocellular carcinoma [12–20], (“The Cancer Genome Atlas” NCI and NHGRI, accessed 7/3/17). SOX10 expression is found in normal breast tissue and up to 40% of breast carcinoma, with enrichment in the unclassified triple-negative and metaplastic carcinomas [21]. Furthermore, SOX10 increases stem/progenitor activity in mammary cells, and SOX10 overexpression causes these cells to undergo a mesenchymal transition [22].

Interestingly, SOX10 expression is required for efficient therapeutic targeting of the activating BRAFV600E mutation in melanoma. This BRAF mutation is found in approximately 50% of patients with advanced melanoma and causes constitutive activation of the Mitogen Activated Protein Kinase (MAPK) pathway [23–27]. Targeted inhibition of the BRAFV600E mutation with the small molecule inhibitor PLX4032 (Vemurafinib) decreases MAPK pathway signaling and has shown rapid responses in patients [28]. However, this agent is rarely curative, due to acquired resistance through several mechanisms employed by tumor cells to increase MAPK signaling in the presence of inhibitor [29–33]. Loss of SOX10 was shown to increase inhibitor resistance via elevated expression of the receptor tyrosine kinase EGFR [34–36]. This suggests SOX10 can regulate EGFR levels in melanoma, and that reducing SOX10 protein may play an important role in acquired resistance.

SOX10 belongs to the SOXE subgroup of proteins, along with SOX8 and SOX9. SOXE proteins function in many diverse cellular processes, including skin and kidney development, neural crest development, chondrogenesis, stem cell reprograming and differentiation [37–39]. Data are emerging to suggest that the varied functions and stability of SOXE proteins may be post-translationally modified by phosphorylation, as has been shown for other transcription factors [40,41]. SOX9 has two cAMP-dependent protein kinase A phosphorylation sites (S64, S211) that increase DNA binding, promoter transactivation, and nuclear localization [42,43]. In addition, SOX9 is phosphorylated by TGF-β at S211, which increases protein stability in chondrogenic cells [44]. However, these three residues are not conserved in SOX10, and only one appears in SOX8, suggesting distinct phosphorylation sites may occur among SOXE proteins [37,45].

To date, very little is known about SOX10 post-translational regulation. In this study, the proteasomal inhibitor MG132 increased SOX10 protein levels and mass spectroscopy identified SOX10 post-translational modifications, consistent with SOX10 protein regulation via phosphorylation events that trigger degradation by the ubiquitin-proteasome system (UPS). Generation of mutants at amino acids S24, S45 and T240, each located in predicted MAPK/CDK binding motifs, allowed investigation of their effect on SOX10 transcription activity, subcellular localization, and stability in melanoma cells. These data extend our knowledge of SOX10 protein regulation, providing important information for identification of molecular pathways that could modulate SOX10 protein levels and contribute to improved melanoma therapy.

## Materials and methods

### Cell culture, transfection and reporter assays

MeWo, NIH3T3 and HeLa cell lines were purchased from ATCC (Manassas, VA) and the 501mel cell line was a generous gift from Dr. Yardena Samuels (The Weizmann Institute of Science, Rehovot, Israel). Cell lines were maintained at 37°C with 5% CO_2_ in DMEM (NIH3T3, HeLa), EMEM (MeWo) or RPMI (501mel) supplemented with 10% FBS and 2 mM L-glutamine (Invitrogen). To transfect cell lines, cells were seeded into 6-well culture plates and transfected 1 day later with 1μg plasmid DNA, complexed with 3ul Lipofectamine2000 reagent (Invitrogen). For luciferase reporter assays, HeLa or NIH3T3 cells were seeded into 24-well culture plates, then co-transfected with 400 ng pMITF2256-Luc [46], 400 ng Tyr-Luc [1–8,47] or 400 ng HuDCT-Luc [9–11,48]; 400 ng WT or phospho-mutant *SOX10-pLenti6*.*2/SOX10-pcDNA3*.*1*; 400ng *MITF-pFLAG* [12–20,48] or *PAX3*-pCEV plasmid [21,46]; and 8 ng pRL-Renilla luciferase plasmid (Promega). Cells were cultured for 48 hours before lysis, and extracts were assayed for luciferase activity using the Dual-Luciferase Reporter Assay System (Promega) using a Fluoroskan Ascent FL Fluorometer (Thermo Fisher Scientific, Waltham, MA). All experiments were carried out in triplicate.

### SOX10 immunoprecipitation and mass spectrometry

501mel melanoma cells were seeded in 150 cm culture dishes 2 days prior to harvest, and cells were treated with 20 μM MG132 proteasomal inhibitor (Sigma, St. Louis, MO) 20 hours before harvest. Cells were rinsed with cold 1x PBS, lysed in 1 mL cold IP buffer (150 mM NaCl, 10 mM Tris-HCL, 1 mM EDTA, 1% triton X100, 0.5% NP-40, 1 mM NaF, 1 mM Na_3_VO_4_, 1 mM PMSF, 1 Roche PIC tablet/10 mL buffer) with constant agitation for 20 minutes at 4°C. Cells were scraped from the dish, subjected to brief sonication at 4°C for 5 seconds, then microfuged 5 mins at 7,000 rpm to remove cellular debris. The supernatant was collected as immunoprecipitation (IP) input, and applied to 200ul Dynabeads Protein G magnetic beads (Life Technologies, Grand Island, NY) for 1 hour preclearing at 4°C. A magnetic field was used to separate preclear beads, and lysate was removed and split into 2 clean tubes: 1 for IgG negative IP sample with 10 μg R&D IgG Control antibody and 1 for SOX10 IP sample with 10 μg SOX10 monoclonal R&D antibody MAB2864 (R&D Systems, Inc., Minneapolis, MN). Lysate and antibody were incubated overnight with rotation at 4°C. The next day, 50 μl magnetic beads were added to each IP sample for a 2 hour incubation at 4°C. Supernatant was reserved for Western blot analysis, and beads were washed 4 times with 500 μl cold IP buffer without the detergents (150 mM NaCl, 10 mM Tris-HCL, 1 mM EDTA, 1 Roche PIC tablet/10mL buffer). Final elution was performed with 50 mM glycine (pH 2.2) for 3 minutes at room temperature. The eluted lysate was immediately neutralized with 1M Tris (pH 8) in a 1:1 volume to volume ratio. IP samples were separated on 8% tris-glycine gels and bands cut that corresponded to SOX10 protein size.

### In-gel digestion

Protein gel bands were processed following a standard in-gel digestion protocol. Briefly, gel bands were minced and destained using 50% acetonitrile in 50 mM ammonium bicarbonate. Proteins were reduced with 10 mM DTT at 56°C, followed by alkylation with 55 mM iodoacetamide at room temperature in the dark. Trypsin digestion was carried out overnight at 37°C with gentle shaking. Peptides were extracted using 1% trifluoroacetic acid in 50% acetonitrile. Samples were vacuum concentrated to dryness and reconstituted in 0.1% formic acid for subsequent liquid chromatography tandem mass spectrometry (LC-MS/MS) analysis.

### LC-MS/MS analysis

LC-MS/MS was performed on a Dionex UltiMate 3000 nano HPLC system coupled online to an Orbitrap Fusion tribrid mass spectrometer (Thermo Scientific). In brief, tryptic peptide mixture was loaded onto a PepMap C18 nano-trap column (Dionex) for 8 minutes at a flow rate of 6.0 μL/min. The peptides were then separated on a 25 cm PicoFrit BetaBasic C18 analytical column (New Objective) with an 80 minutes linear gradient (5–35% acetonitrile in 0.1% formic acid) at a flow rate of 300 nL/min. Eluted peptides were ionized using electrospray ionization in positive ion mode and detected in the mass spectrometer. Precursor ions were selected for MS/MS using a data-dependent method in which the most intense ions from the MS1 precursor scan were sequentially fragmented within a 3 second cycle time. All precursor ions were measured in the Orbitrap with the resolution set at 60,000. Precursor ions were fragmented by higher energy collision-induced dissociation at a normalized collision energy of 35%, and all fragment ions were measured in the linear ion trap.

### Peptide and protein identification

All LC-MS/MS data were searched using the Sequest algorithm within Proteome Discoverer 1.4 (Thermo Scientific) against the human Swiss-Prot protein sequence database to obtain peptide and protein identifications. For all searches, trypsin was specified as the enzyme for protein cleavage allowing up to 2 missed cleavages. Oxidation and phosphorylation were selected as dynamic modifications while carbamidomethylation was set as a fixed modification. Mass tolerances of 20 ppm and 0.8 Da were set for precursor and fragment ions, respectively. For MS/MS data visualization and further validation of identified phosphopeptides, Sequest results were imported into Scaffold (Proteome Software). False discovery rates were set at 1% for both peptide and protein identifications. Spectra of phosphopeptides were manually inspected to confirm phosphorylation site assignments.

### Cloning SOX10 phospho-mutant constructs

Mammalian expression vectors containing wild type (WT) *SOX10* cDNA were made using Gateway technology to recombine a pDonr221-*SOX10* donor plasmid with a pLenti6.2/V5 destination vector (Invitrogen, Carlsbad, CA) or pcDNA3.1 destination vector (Invitrogen, Carlsbad, CA). *SOX10* phospho-mutant plasmids were generated using an overlapping two-fragment PCR amplification strategy. Forward and reverse primers to mutate Ser or Thr to Ala were as follows: S24A forward 5’- GAGGAGCCCCGCTGCCTGGCCCCGG-3’ and reverse 5’- CCGGGGGCCAGGCAGCGGGGCTCCTC-3’, S45A forward 5’- GGCCTGCGAGCCGCCCCGGGG-3’ and reverse 5’- CCCCGGGGCGGCTCGCAGGCC-3’, T240A forward 5’- ATGGCCCACCCGCCCCTCCAACCA-3’ and reverse 5’- TGGTTGGAGGGGCGGGTGGGCCAT-3’ (IDT, Coralville, IA). These primers were used in 2 PCR reactions with WT *SOX10*-pLenti6.2 plasmid template and either 5’ or 3’ SOX10 primers. 5’ and 3’-*SOX10* PCR-containing fragments were gel purified before using together as template for full length *SOX10* using ATTB1-SOX10 forward and ATTB2-SOX10 reverse primers. Gateway BP PCR products were inserted into pDonr221 entry vector, sequence verified, and subsequently transferred into pLenti6.2/V5 using standard Gateway protocols (Invitrogen). Clones were prepared by Qiagen EndoFree Maxiprep kit (Qiagen, Germantown, MD).

### Immunoblotting and cycloheximide pulse-chase assays

Protein gels and Western blots were performed using standard protocols. Primary antibodies were: monoclonal SOX10 (R&D Systems #MAB2864, Minneapolis, MN), monoclonal alpha-Tubulin (Calbiochem cat# CP06), monoclonal GAPDH (Santa Cruz cat# sc-47724) and monoclonal anti-V5 antibody (Invitrogen cat# R960-25). HRP-conjugated secondary antibody was obtained from Jackson ImmunoResearch Laboratories (West Grove, PA). Cycloheximide pulse chase was performed in 501mel and MeWo melanoma cells transfected with WT or phospho-mutant SOX10 constructs tagged with an N-terminal V5 epitope tag, followed by treatment with 100ug/mL cycloheximide 48 hours post-transfection. Cells were rinsed once with 1x PBS, harvested by scraping into 2x SDS sample buffer (Invitrogen cat# LC2676), sonicated and boiled before quantification of protein in Qubit fluorometer (Invitrogen). Cell lysates for each construct were harvested at the beginning of the time course (0 hour, no cycloheximide added), then at several time points after addition of cycloheximide to the culture. For Western blot analysis, 20 μg of protein was loaded onto 8% tris-glycine gels, protein transferred onto PVDF membranes via semi-dry transfer apparatus (BioRad), then membranes were blocked with 5% non-fat dry milk in 1x TBST (Tris-buffered saline, 0.1% tween20) for 1 hour before overnight incubation in primary antibody at 4°C. Blots were washed 4 times in 1x TBST before a 1 hour incubation in secondary antibody diluted into block. Developed membranes were scanned and densitometry performed with ImageJ 1.47t software (https://imagej.nih.gov, NIH, Bethesda, MD).

### Immunohistochemistry

HeLa cells and 501mel melanoma cells were seeded into 8-well chamber CC2-coated slides (Thermo Fisher Scientific) one day before staining. Cells were rinsed with 1X PBS, fixed in 4% paraformaldehyde for 10 minutes, rinsed briefly with 1X PBS 0.1% Tween, then permeabilized with 0.1% Triton for 10 minutes. Following 30 minute block in 1mg/mL BSA (Sigma cat.# A3059), cells were incubated 2 hours with primary antibodies in block (anti-SOX10, Santa Cruz cat.# sc-17342; anti-V5, Invitrogen cat.# 46–0705), then rinsed and incubated 20 minutes in Alexa 488 or 568 secondary antibodies (Invitrogen) diluted in block. Following a 48 hour incubation, cells were fixed and stained to visualize their subcellular localization. Cells were rinsed before mounting with ProLong Gold mounting media with DAPI (Invitrogen). Cell images were taken on Zeiss AxioImager.D2 upright microscope with AxioVision 4.8 software (Carl Zeiss Microscopy, Thornwood, NY).

## Results

### SOX10 protein is degraded by the UPS

To determine if SOX10 protein levels are regulated by the UPS, 501mel cells were treated with the proteasome inhibitor MG132. This resulted in SOX10 accumulation, indicating SOX10 protein degradation is mediated by the proteasome (S1 Fig). The UPS system relies on polyubiquitination of targets to degrade proteins, usually preceded by phosphorylation of specific residues along the protein [22,49]. Therefore, we sought to evaluate SOX10 post-translational modifications in melanoma cells.

### SOX10 phosphorylation sites identified by mass spectrometry

**Fig 1:**
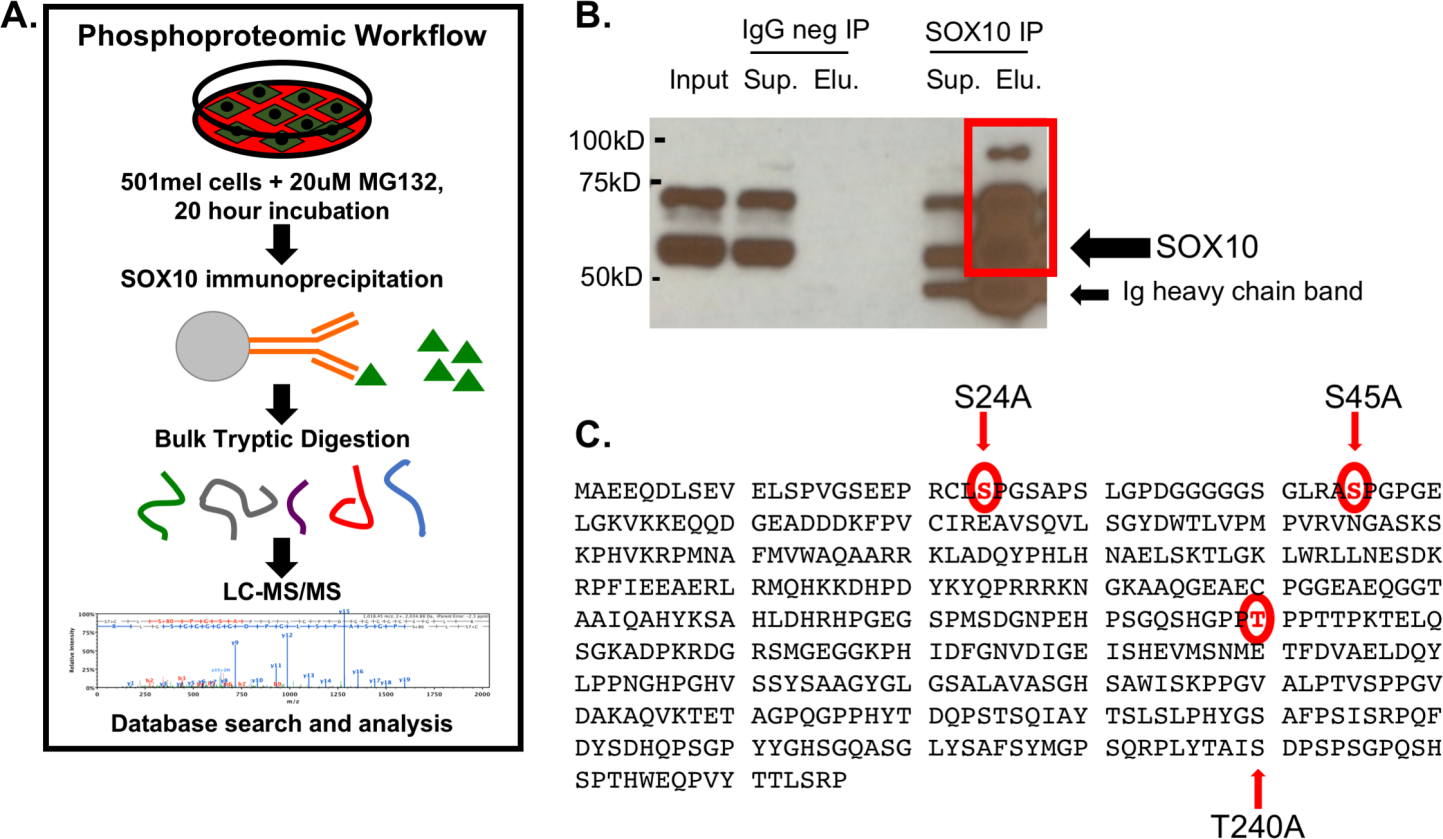
Mass spectrometry analysis identifies SOX10 phosphorylation sites. A. Workflow schematic of SOX10 protein analysis by mass spectrometry. 501mel cells were treated with MG132 proteasomal inhibitor before scraping cells and performing immunoprecipitation (IP) using SOX10 antibody to isolate protein. The eluted proteins were separated by SDS-page, followed by staining and removal of bands corresponding to 55kD, 75kD and 100kD. All three gel bands were subjected to destaining, in-gel digestion and extraction before running LC-MS/MS. B. Portions of IP samples were separated on SDS-page gel, followed by transfer onto PVDF membrane and Western blotting to confirm SOX10 isolation in the eluted samples being used for mass spectrometry. C. The three phosphorylation sites selected for mutation and characterization are shown in the context of full length SOX10 (Genbank ID NM_006941).

To increase SOX10 protein levels and allow for identification of potential SOX10 post-translational modifications, 501mel cells were treated with MG132 prior to isolation of SOX10 by immunoprecipitation (Fig 1A and 1B). Three intense bands of 55kD, 70kD, and 100kD were detected by Western blot of SOX10 IP-eluted sample (Fig 1B), with the most abundant band at 55kD, the predicted size for unmodified SOX10 protein. As the larger bands could represent post-translationally modified SOX10 protein, all three bands were individually isolated, digested and analyzed by mass spectrometry.

**Fig 2:**
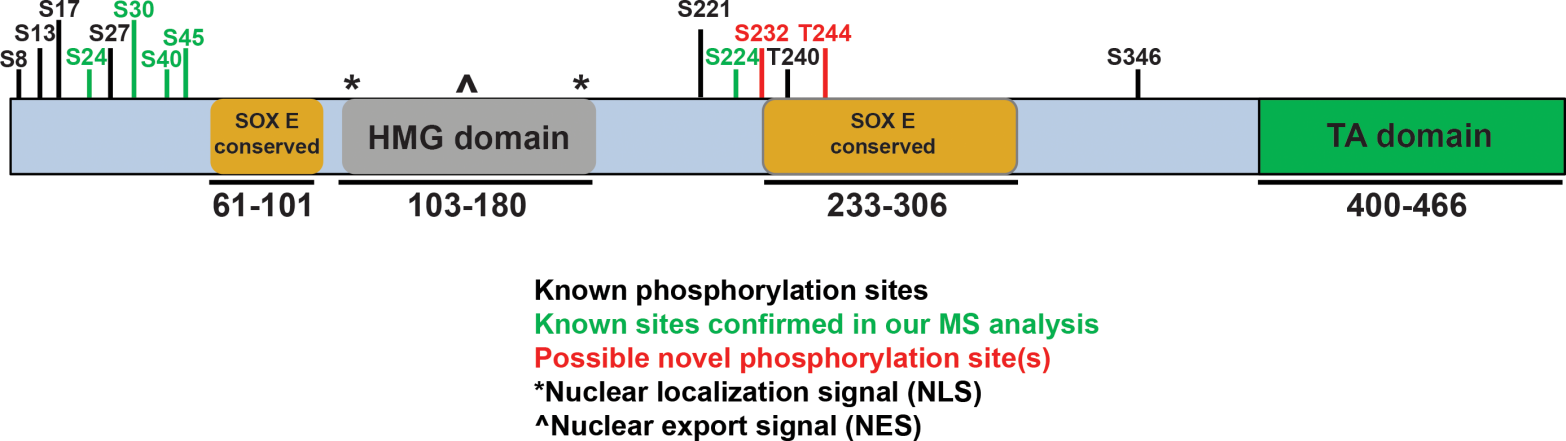
SOX10 post-translational modifications identified in MG132-treated 501mel cells. This schematic representing the SOX10 protein indicates known domains, SOXE conserved regions, and phosphorylated residues, as follows: black bars show known phosphorylation sites, green bars show known sites that were confirmed in this study, and red bars show novel sites from this study. The phosphorylated residues S224, S232, T240 and T244 were observed on numerous peptide fragments, and one or all four are plausible; their close proximity and the limited fragmentation capability in the digest restrict more precise determination among these residues. The nuclear localization and nuclear export signal regions are unaffected by the phosphorylation sites.

Mass spectrometry analysis revealed 13 unique SOX10 peptides with 37% amino acid coverage in the 55kD band, and 3 unique SOX10 peptides with 14% amino acid coverage in the 70kD band. No SOX10 peptides were identified in the 100kD band. From the 55kD and 70kD SOX10 IP samples, a total of seven amino acids exhibited post-translational phosphorylation (Fig 2, S1 Table). Two of these phosphorylation sites were potentially novel (S232, T244), while the remaining five confirmed previous mass spectroscopy studies, overlapping phosphorylated residues previously identified in melanoma tissue (S24, S30, S45), breast tumors (S24, S30, S45, S232) and mouse neuroblastoma (S40) [23–27,50–53]. Of note, the SOX10 phosphorylation sites discovered here and in previous studies occured in two distinct clusters (Fig 2), one at the amino terminus 5’ of the SOXE conserved dimerization domain, and the other in the center of the protein, partially overlapping with the 5’ end of an additional SOXE conserved domain [28,54–56]. Analysis of the peptide spanning amino acids 216 to 246, identified in both the 55kD and 70kD IP samples, suggested multiple phosphorylation modifications. Scaffold analysis (Scaffold_4.7.2 Proteome Software, Inc.) assigned these phosphorylation events to amino acids S224, S232 and T244. Examination of ion spectrums suggested phosphorylation modification could occur on any of these three residues, in addition to the T240 residue found to be phosphorylated in breast tumor samples [29–33,52]. Although tryptic digestion did not produce fragments small enough to permit exact assignment of the phosphorylated residues between S224 and T244 (as is common in mass spectroscopy experiments), analysis of the ion spectrum of each individual fragment determined residue T240 had the highest likelihood of phosphorylation, bringing the total number of phosphorylated SOX10 residues identified in this study to eight.

### MAPK and CDK motifs mark regions of SOX10 phosphorylation

Identified phosphorylation sites were further analyzed using the Eukaryotic Linear Motif (ELM) database (ELM 2016-data, http://elm.eu.org), to assess if observed phosphorylation sites resided within predicted functional protein motifs corresponding to defined kinases. This analysis identified multiple Class IV WW domains, which are MAPK and CDK target sites, overlapping with the S24, S45 and T240/T244 phosphorylation sites. The presence of phosphorylation sites within these domains were of particular interest because of the importance of the MAPK pathway in melanoma progression, and the potential involvement of SOX10 protein in acquired resistance to MAPK inhibitors. Based on these overlapping WW domains and predicted phosphorylation events, SOX10 constructs containing an N-terminal V5 epitope tag were generated with the following mutations: S24A, S45A, double mutant S24A,S45A, and T240A (Fig 1C).

### SOX10 phosphorylation mutant proteins localize to the nucleus

**Fig 3:**
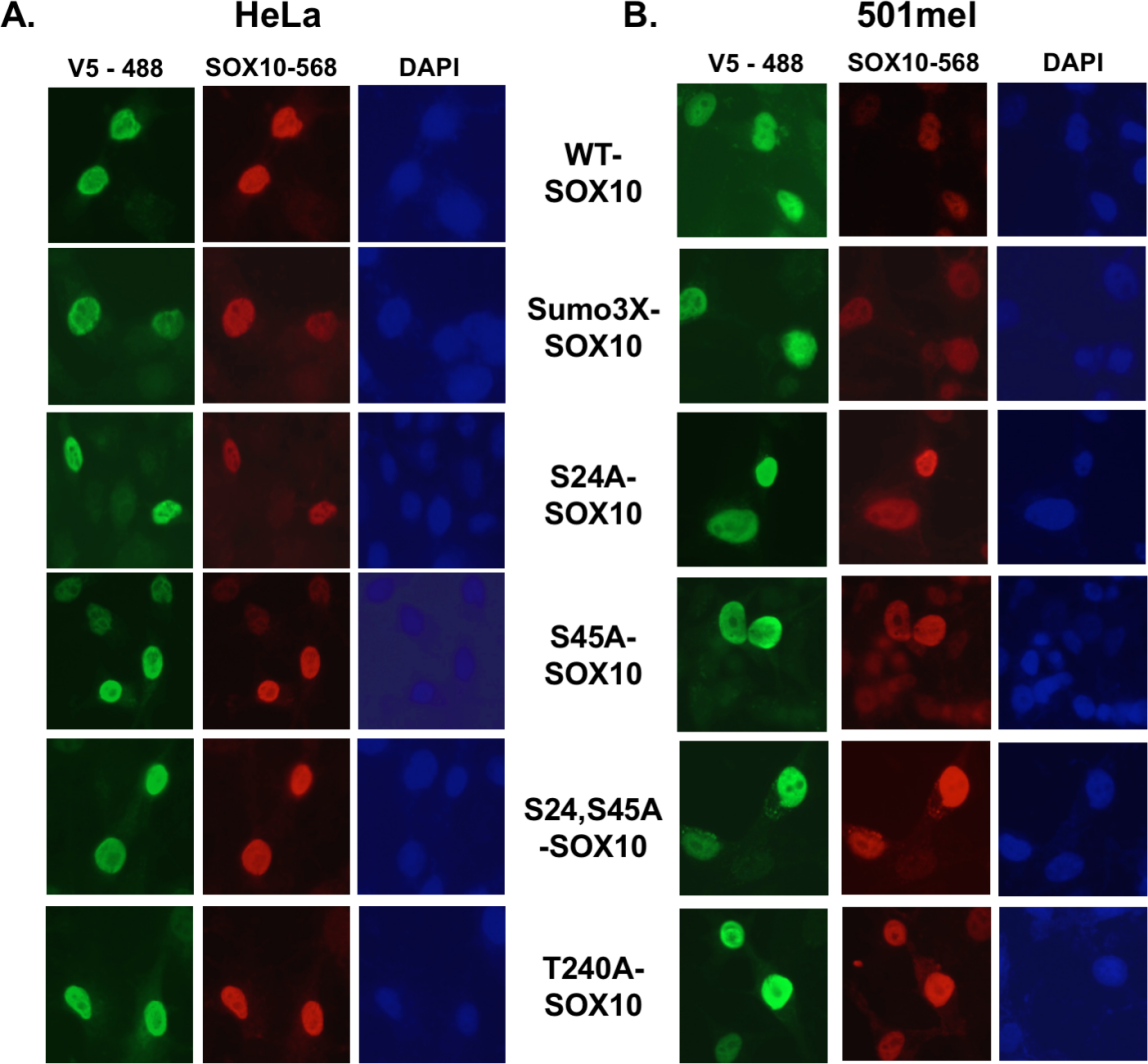
SOX10 phosphorylation mutants retain nuclear localization. A,B. HeLa cells (A) and 501mel melanoma cells (B) were transfected with WT and phospho-mutant SOX10 constructs, and after 48 hours were fixed and stained to visualize subcellular localization of WT SOX10 and SOX10 phosphoryation mutant proteins. The Sumo3x SOX10 mutant was used as a post-translational modification control, as it is known to express in the nucleus despite mutations in all 3 sumoylation sites. No differences in localization are seen in the SOX10 phosphorylation mutants relative to WT SOX10. The V5 antibody (V5-488) stains exogenous SOX10 in both cell lines, while the SOX10 antibody (SOX10-568) stains both exogenous and endogenous SOX10 in 501mel cells (HeLa cells do not express endogenous SOX10).

SOX10 functions as a transcription factor and primarily localizes to the cell nucleus, where it binds target promoters and enhancer elements to regulate gene expression. To assess the impact of SOX10 phosphorylation on subcellular localization, the WT and phospho-mutant SOX10 constructs were overexpressed in HeLa and 501mel cell lines. Staining for either SOX10 protein or the V5 epitope showed nuclear staining of all phospho-mutants in both cell lines that was indistinguishable from WT SOX10 (Fig 3), demonstrating that mutation of these phosphorylation sites does not significantly impact expression levels or nuclear localization of SOX10 protein.

### SOX10 phospho-mutant proteins activate target promoters

**Fig 4:**
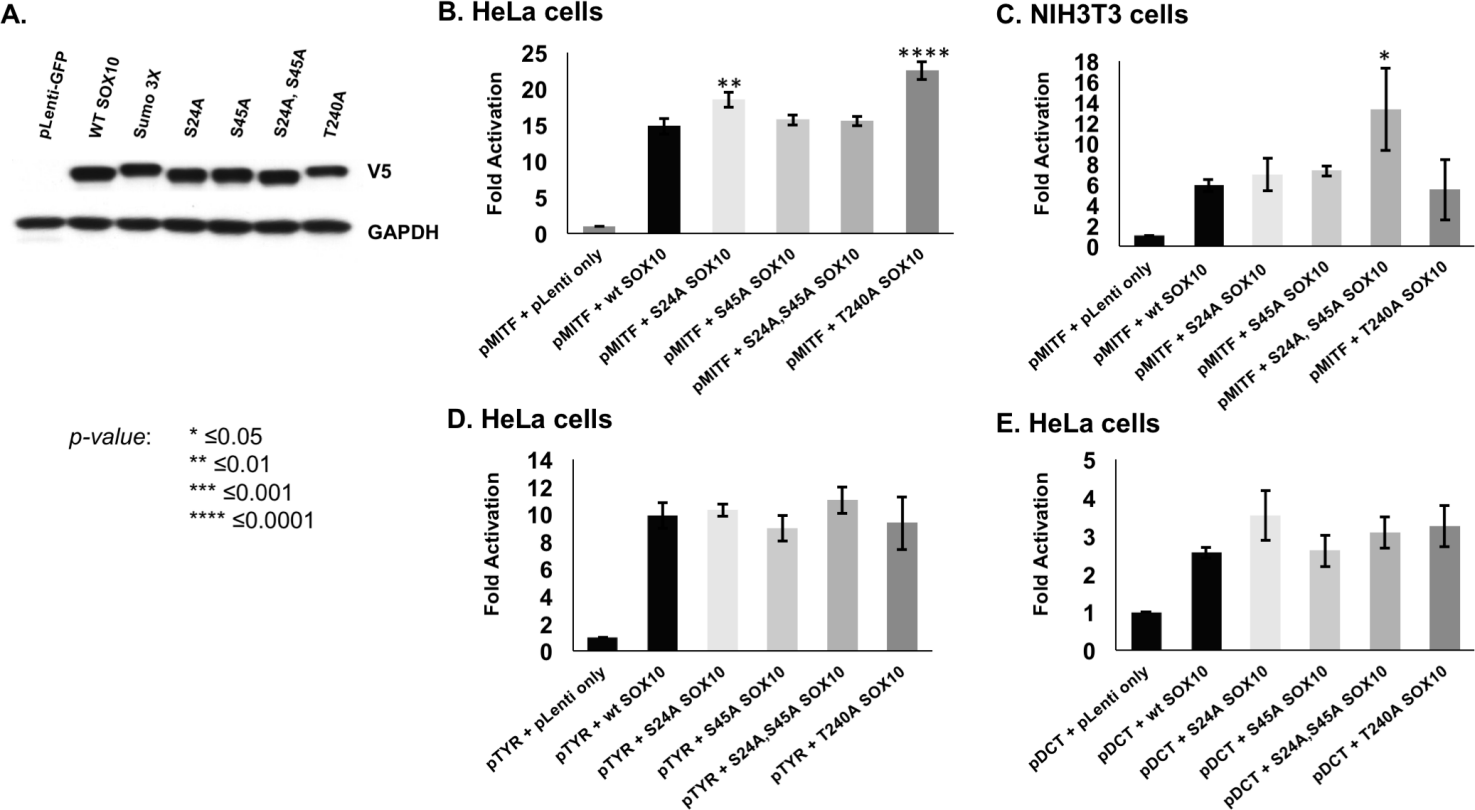
SOX10 phospho-mutants exhibit cell-specific differences in activation of the MITF promoter. A. Over-expression of WT and phospho-mutant SOX10 yields similar protein levels in HeLa cells at 48 hours on Western blot. SOX10 phospho-mutants show bands running at slightly different sizes; the SOX10 Sumo3x mutant is included as a control for protein band shifting that results from altering amino acid residues at sites of post-translational modifications. B,C. Representative luciferase data showing activation of p*MITF* from WT and SOX10 phospho-mutants in HeLa (B) and NIH3T3 cells (C); the S24A and T240A constructs showed significantly greater promoter activation in HeLa cells, while the S24A, S45A construct showed significantly greater promoter activation in NIH3T3 cells. Replicate data sets for p*MITF* can be seen in S2 Fig. D. p*TYR* promoter luciferase data showed no significant differences between phospho-mutants and WT SOX10 in HeLa cells; representative dataset is shown. E. p*DCT* promoter luciferase data showed no significant differences between phospho-mutants and WT SOX10 in HeLa cells; representative dataset is shown. Statistics were calculated using one-way ANOVA with Bonferroni’s multiple comparison test, three independent assays per promoter construct.

SOX10 alone or in combination with known binding partners activates transcription of multiple genes driving melanocyte differentiation and cell function [34–36,57–61]. We evaluated the ability of each SOX10 phospho-mutant to transactivate well-characterized melanocyte gene promoters, alone or synergistically in combination with paired box 3 (PAX3) and microphthalmia-associated transcription factor (MITF), using luciferase constructs driven by the human *MITF*, *TYR* and *DCT* promoters [37–39,47,57,58]. Western blots confirmed that all mutant constructs were expressed in HeLa cells at similar levels (Fig 4A). A SOX10 Sumo3x mutant, in which all three SOX10 sumolyation consensus motifs are mutated (K55A, K246A, K256A), was used to demonstrate SOX10 Western band shifting that occurs from lost post-translational modifications [40,41,62]. Similarly, the SOX10 phospho-mutants showed moderate band size shifting, with S24A and S45A mutant proteins running smaller than WT SOX10 and the T240A mutant protein running larger (Fig 4A).

A subset of the SOX10 phospho-mutant constructs showed moderate but significantly increased activity on the *MITF* promoter (p*MITF*) compared to WT SOX10 protein activation in HeLa (Fig 4B, S2 Fig) and NIH3T3 cells (Fig 4C, S2 Fig). We observed distinct and different responses, depending on the cell line context. The S24A and T240A mutant proteins showed significantly increased activation of p*MITF* in all biological replicates in HeLa cells, while in NIH3T3 cells only the S24A, S45A double mutant construct showed increased activation in comparison to WT SOX10 (Fig 4B and 4C, S2 Fig). When PAX3 was co-expressed in combination with WT and phospho-mutant SOX10 constructs in HeLa cells, no significant reproducible differences were observed (S3 Fig).

No consistently significant differences from WT SOX10 were observed in the abilities of the phospho-mutants to transactivate either *TYR* or *DCT* promoters when these promoters were assayed alone (Fig 4D and 4E) or in combination with MITF (S3 Fig). Taken together, these data suggest cell-specific context effects on SOX10 transcriptional activity via these phosphorylation sites. These results also indicate that the S24A, S45A, and T240A SOX10 phosphorylation sites are not ubiquitously utilized to regulate transcriptional activity of SOX10 protein, and do not alter SOX10’s synergistic activation with PAX3 and MITF on target promoter regions.

### Phospho-mutant SOX10 proteins show variable stability

**Fig 5:**
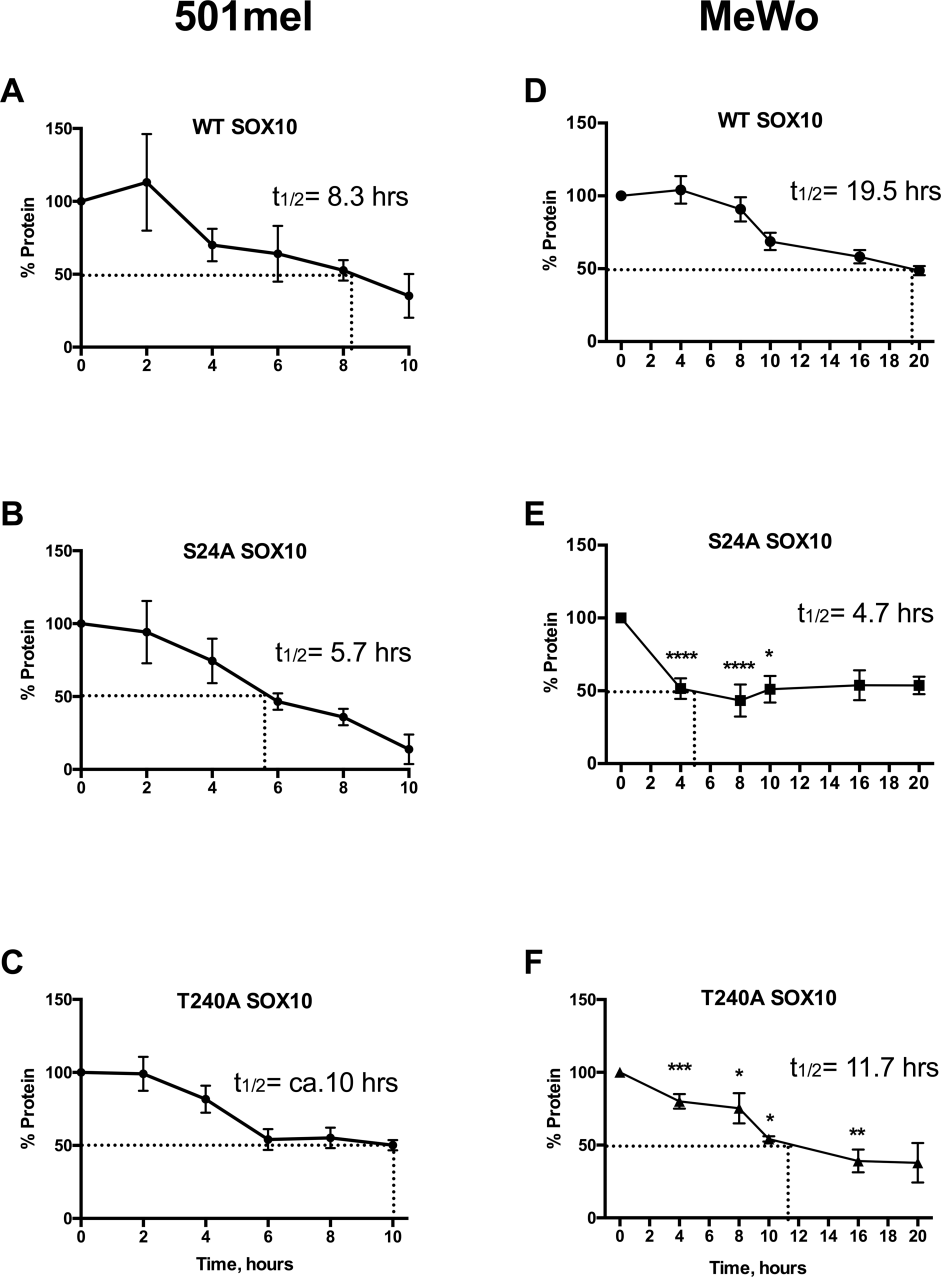
Mutation of SOX10 phosphorylation sites causes distinct changes in protein stability. Cycloheximide pulse-chase assays in 501mel (A-C) and MeWo (D-F) cells revealed altered stability of SOX10 phospho-mutants compared to WT SOX10 protein. A. WT SOX10 showed a half-life of 8.3 hours in 501mel cells. B,C. Stability of SOX10 phospho-mutants S24A and T240A is not significantly different from WT SOX10 in 501mel cells (two-way ANOVA, p = 0.25). D. WT SOX10 stabilty in MeWo cells exhibited a half-life of 19.5 hours. E. S24A SOX10 mutant protein showed reduced stability in MeWo cells with a half-life of 4.7 hours. F. T240A SOX10 mutant protein showed reduced stability in MeWo cells with a half-life of 11.7 hours. Both the S24A and the T240A mutant proteins exhibited significant differences relative to WT SOX10 protein in MeWo cells (two-way ANOVA, p = 0.0057 for protein type, p<0.0001 for time and interaction); by Bonferroni’s multiple comparisons post-test, these differences were significant for SOX10 S24A from 4 hours through 10 hours, and were significant for SOX10 T240A from 4 hours through 16 hours (P-values: *≤0.05, **≤0.01, ***≤0.001, ***≤0.0001). Data are compiled from 3 independent assays, with standard deviations plotted.

Specific phosphorylation or dephosphorylation of amino acid residues can change protein stability, as has been documented for SOX9 [42–44]. To investigate the stability of SOX10 phospho-mutants, cycloheximide pulse-chase analysis was performed using WT and phospho-mutant SOX10 constructs that were overexpressed in 501mel and MeWo cells. The half-life of WT SOX10 was 8.3 hours in 501mel cells and 19.5 hours in MeWo cells (Fig 5A–5D), in contrast to the previously reported 6 hour half-life of SOX10 overexpressed in both HeLa cells [7,44] and Neuro2A neuroblastoma cells [37,45,55]. All 4 SOX10 phospho-mutant proteins were analyzed in 501mel cells. While they were not significantly different from WT SOX10 protein (two-way ANOVA), the protein half-lives and degradation rates showed notable trends towards the mutants altering protein stability (Fig 5A–5C). Mutation of the S24 phosphorylation site showed a more rapid degradation, with a half-life of 5.7 hours when mutated alone, and a half-life of 5.8 hours when mutated in combination with S45 (Fig 5B, S4 Fig). Mutation of the S45 residue alone showed a slightly shorter half-life of 7 hours (S4 Fig). Conversely, the T240A mutant protein exhibited a notable plateau of SOX10 expression, as expression levels neared 50% from 6 hours to 10 hours (Fig 5C).

The S24A and T240A SOX10 mutant constructs were also analyzed by cycloheximide pulse chase analysis in MeWo cells (Fig 5D–5F). In this cell line, both the S24A and the T240A mutant proteins exhibited significant differences in degradation relative to WT SOX10 protein (two-way ANOVA, p = 0.0057). Even in the context of a longer 19.5 hour half-life for WT SOX10, the S24A and the T240A SOX10 phospho-mutant protein had reduced stability, with half-lifes of 4.7 and 11.7 hours, respectively. In MeWo cells, the S24A and T240A SOX10 mutants again demonstrated a SOX10 degradation plateau near 50%. These data suggest that SOX10 protein regulation is complex and may involve feedback mechanisms for protein regulation. Similar to the luciferase assays, these data suggest cell-specific context effects on SOX10 stability via these phosphorylation sites.

## Discussion

Transcription factor function is precisely regulated at both the mRNA and protein level, and post-translational phosphorylation is one mechanism that governs transcription factor activity [40,41,46]. Furthermore, cancer progression may employ altered phosphorylation of pivotal transcription factors, as has been suggested for MITF, PAX3, and β-catenin in melanoma [63–65]. Therefore, understanding post-transcriptional regulation of SOX10 via phosphorylation may be crucial to developing effective anti-melanoma therapies. This study identifies eight SOX10 protein phosphorylation sites by mass spectrophotometry in melanoma cells. Previously, four large scale proteomic screens provided evidence of SOX10 modifications, however functional relevance of these SOX10 protein modifications were not assessed [50–52,66]. Data from this study confirms the previously identified phosphorylation sites of S24, S30, S40, and S45. In addition, this analysis shows S224 and T240 to be phosphorylated in melanoma cells, whereas previously both were only identified in breast cancer-derived cells. Furthermore, this study identifies potentially novel SOX10 phosphorylation events at S232 and T244.

Integration of the SOX10 phosphorylation sites discovered in our mass spectroscopy analysis with those identified in previous studies in breast cancer, neuroblastoma and melanoma cells indicates that SOX10 phosphorylation occurs in two distinct clusters close to SOXE-conserved domains [50–52,66,67] (Fig 2), highlighting two regions available for protein-protein interactions leading to post-translational modifications. Interestingly, these two clusters overlap with those identified in other SOXE proteins (S5 Fig). The first cluster of phosphorylated residues (S8 to S45) resides at the amino terminus just 5’ to the SOXE conserved dimerization (DIM) region, which functions in formation of homo- and heterodimers [54,56]. The second cluster (S221 to T244) resides between the HMG domain and partially overlaps another SOXE conserved region (the K2 region) [55]. The HMG domain regulates DNA binding and also interacts with multiple other transcriptional regulators, while the K2 domain is suggested to mediate tissue-specific transactivation functions [39,55,68]. Of note, Clustal analysis of SOX8, SOX9, and SOX10 [69] found that five out of eight of the SOX10 phosphorylated residues (S24, S30 and S232, T240 and T244) are highly conserved, showing potential for serine/threonine phosphorylation among all three SOXE family members. The serine/threonine conservation at these residues suggests similar regulatory pathways could control all SOXE proteins, while the remaining non-conserved sites could indicate individual post-translational regulation pathways for each SOXE protein. Overall, these SOXE phosphorylated regions suggest common structural accessibility and functional significance, with the potential for both overlapping and unique regulation of each SOXE protein within these regions.

The SOX10 phosphorylated residues S24, S45 and T240 were selected for further evaluation with respect to DNA binding, transactivation, subcellular localization, and stability because they reside within WW binding domains, which are predicted targets of proline-directed kinases such as MAP kinases and cyclin-dependent kinases [70], both critical pathways for melanoma proliferation and progression. Since S45 is unique to SOX10, it thus holds the potential for regulation that is distinct from other SOXE family members. No alterations in protein localization were detected for the mutant proteins, however this is not unexpected as none are located within previously characterized SOX10 nuclear localization and nuclear export protein domains [71,72].

We found a modest increase in activity of the S24A and T240A SOX10 phospho-mutant proteins on the *MITF* promoter in HeLa cells (1.3- to 2.1- fold over WT SOX10). However, no reproducible differences were apparent in the transactivation ability of WT and SOX10 phospho-mutant proteins on the proximal promoters of other well-characterized SOX10 target genes or with synergistic cofactor expression. We also found a small but significant increase in activity of the S24A, S45A mutant on the *MITF* promoter in NIH3T3 cells (1.5–2.3-fold over WT SOX10). The results of these *in vitro* assays do not exclude the possibility that these alterations may affect other SOX10 functions, such as SOX10’s ability to regulate gene expression at other loci or modulate interactions with other transcriptional cofactors. For example, these sites may regulate interactions between SOX10 and other chromatin regulators. SOX10 has the capacity to bend DNA [73], binds predominately at distal enhancers rather than promoters [74], and has demonstrated interactions with the BRG1/BAF complex and Chromodomain helicase DNA binding protein 7 [75–77]. Additional functional assessments, performed on a genome-wide scale, may be required to fully dissect the *in vivo* functions of these SOX10 phosphorylation sites.

Alternatively, these sites of phosphorylation modification may regulate protein stability. Analysis of SOX10 protein stability in MeWo cells showed significant changes in SOX10 phospho-mutant stability, pointing out regions by which SOX10 protein levels can be regulated in the cell. While future studies will be required to assess biological significance of these phosphorylation sites *in vivo*, a similar shift in protein stability for another SOXE family member, SOX9, has been implicated in lung cancer metastasis [78]. Additionally, phosphorylation at SOX10 T240 and T244 is consistent with previous data showing GSK3B-dependent SOX10 ubiquitination at this region by FBXW7 E3 ligase, as protein phosphorylation is required prior to addition of the ubiquitin moiety [79]. Our analysis supports and extends this data, identifying T240 phosphorylation in melanoma cells and demonstrating decreased protein stability of SOX10 T240A compared to WT protein, as assayed in MeWo melanoma cells. Melanoma cell lines are notoriously heterogenous in driver mutations, as well as large chromosomal deletions and amplifications which give rise to highly varied genetic backgrounds [27]. The large difference in WT SOX10 stability in the two cell lines used in the cycloheximide analysis is intriguing and suggests modifiers of SOX10 stability exist in different levels among these lines.

This study provides a functional assessment of phosphorylation that occurs on SOX10 protein in melanoma cells, highlighting specific residues that may modulate SOX10 protein levels. This could provide important insight into the regulation of SOX10 protein levels in melanoma cells, and contribute to our understanding of pathways involved in tumor-acquired resistance. It will also be of great interest to determine in future studies if the SOX10 phosphorylation demonstrated here in melanoma can be extended to breast carcinomas and other cancers that maintain SOX10 expression.

## Supporting information

S1 Fig Proteasomal inhibition with MG132 treatment results in accumulation of SOX10 protein. Comparison of cell lysates from pLenti empty vector transfected cells (pLenti) treated with DMSO versus MG132 shows marked increase of endogenous SOX10 protein. Upon transfection of 501mel cells with a SOX10-pLenti construct (pSOX10), SOX10 protein levels are increased in comparison to pLenti under DMSO treatment, and are markedly increased when cells over-express SOX10 in combination with the MG132 proteasomal inhibitor.
(PDF)

S2 Fig Replicate datasets for SOX10 phospho-mutant p*MITF* luciferase assays in HeLa cells and NIH3T3 cells. These independent replicate assays expand on data in Fig 4B and 4C. Activation of the p*MITF* luciferase promoter construct by the S24A and T240A SOX10 phospho-mutants was moderately but significantly increased relative to WT SOX10 in HeLa cells (top panels A and B). Activation of the p*MITF* construct by the S24A, S45A SOX10 double mutation construct was moderately but significantly increased relative to WT SOX10 in NIH3T3 cells (bottom panels C and D). Statistical analysis: one-way ANOVA with Bonferroni’s multiple comparison test.
(PDF)

S3 Fig SOX10 phospho-mutant luciferase assays in HeLa cells with co-expression of the cofactors PAX3 and MITF show no significant differences from WT SOX10. A. Synergistic activation of *pMITF* was achieved by all SOX10 constructs tested when co-expressed with PAX3 protein. B,C. Synergistic activation of p*TYR* (B) and p*DCT* (C) was achieved by all SOX10 phospho-mutant constructs when co-expressed with MITF. While significant differences relative to WT SOX10 were achieved in some individual samples, none of these were consistent across all biological replicates. Statistical analysis: one-way ANOVA with Bonferroni’s multiple comparison test p-value *≤0.05, **≤0.01, ***≤0.0001.
(PDF)

S4 Fig Cycloheximide pulse chase stability data for S45A and S24A, S45A SOX10 mutant proteins in 501mel cells. A. The S45A SOX10 phospho-mutant showed protein degradation similar to WT SOX10, with a half-life of 7 hours. B. Stability data for the S24A, S45A double mutant showed a similar half life as that of the S24A mutation alone, with a half-life of 5.8 hours.
(PDF)

S5 Fig SOXE protein phosphorylation sites cluster in similar pattern. Schematic representation of all 3 SOXE proteins (SOX8, SOX9 and SOX10). Functional domains are highlighted, and phosphorylation sites have been mapped along the length of each protein (Yusuf D, Butland SL, Swanson MI, Bolotin E, Ticoll A, Cheung WA, et al. The Transcription Factor Encyclopedia. Genome Biology 2012 13:3. BioMed Central; 2012 Mar 29;13(3):R24) (PhosphoSitePlus, Cell Signaling). An N-terminal cluster of phosphorylated residues occurs proximal to the SOXE conserved dimerization region in both SOX9 and SOX10, and phosphorylation sites are clustered centrally in all 3 SOXE proteins.
(PDF)

S6 Fig Uncropped blot for Fig 1A. (PDF)

S7 Fig Uncropped blot for S1 Fig. (PDF)

S1 Table SOX10 post-translational modifications identified in mass spectrometry analysis. These include oxidation, phosphorylation and carbamidomethyl binding. Digested peptide sequences are shown, along with each modification identified within that length of amino acids. The XCorr value is the cross-correlation value from the database search; values above 2.0 typically indicate a good correlation with higher values meaning increased correlation. The DCn score is the Delta Correlation value, with numbers above 0.1 indicating good correlation.
(PDF)
