# Supplementary figures and images for: Identification and functional analysis of SOX10 phosphorylation sites in melanoma
Source: PLoS One. 2018 Jan 9;13(1):e0190834. doi: 10.1371/journal.pone.0190834 (PMC5760019; doi:10.1371/journal.pone.0190834)

S1 Fig: Proteasomal inhibition increases levels of SOX10

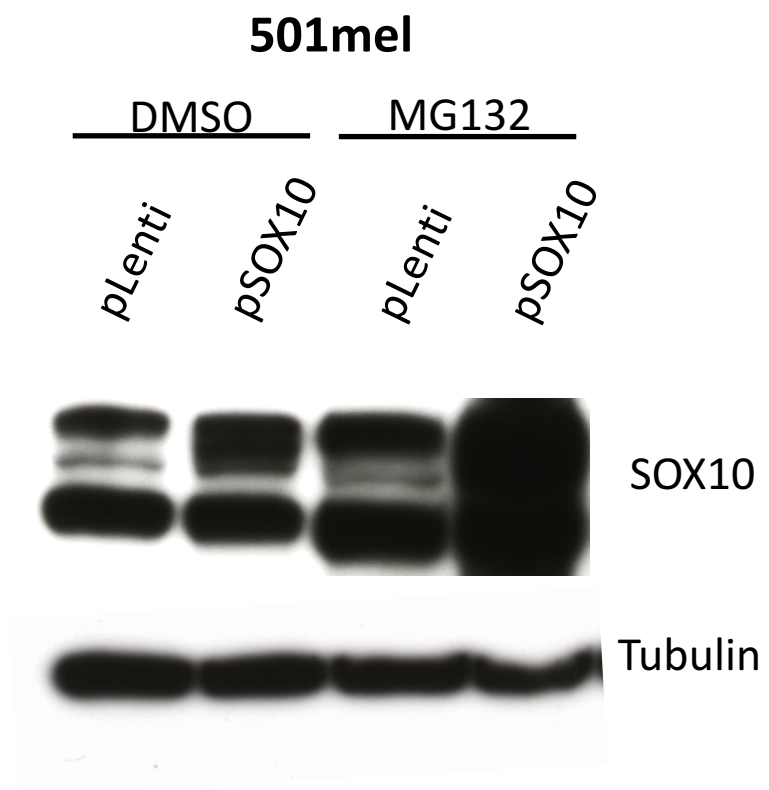

Supplement: S1 Fig — Comparison of cell lysates from pLenti empty vector transfected cells (pLenti) treated with DMSO versus MG132 shows marked increase of endogenous SOX10 protein. Upon transfection of 501mel cells with a SOX10-pLenti construct (pSOX10), SOX10 protein levels are increased in comparison to pLenti under DMSO treatment, and are markedly increased when cells over-express SOX10 in combination with the MG132 proteasomal inhibitor. (PDF) [file pone.0190834.s001.pdf]

S2 Fig: SOX10 phospho-mutant pMITF luciferase assay replicate data sets in multiple cell lines

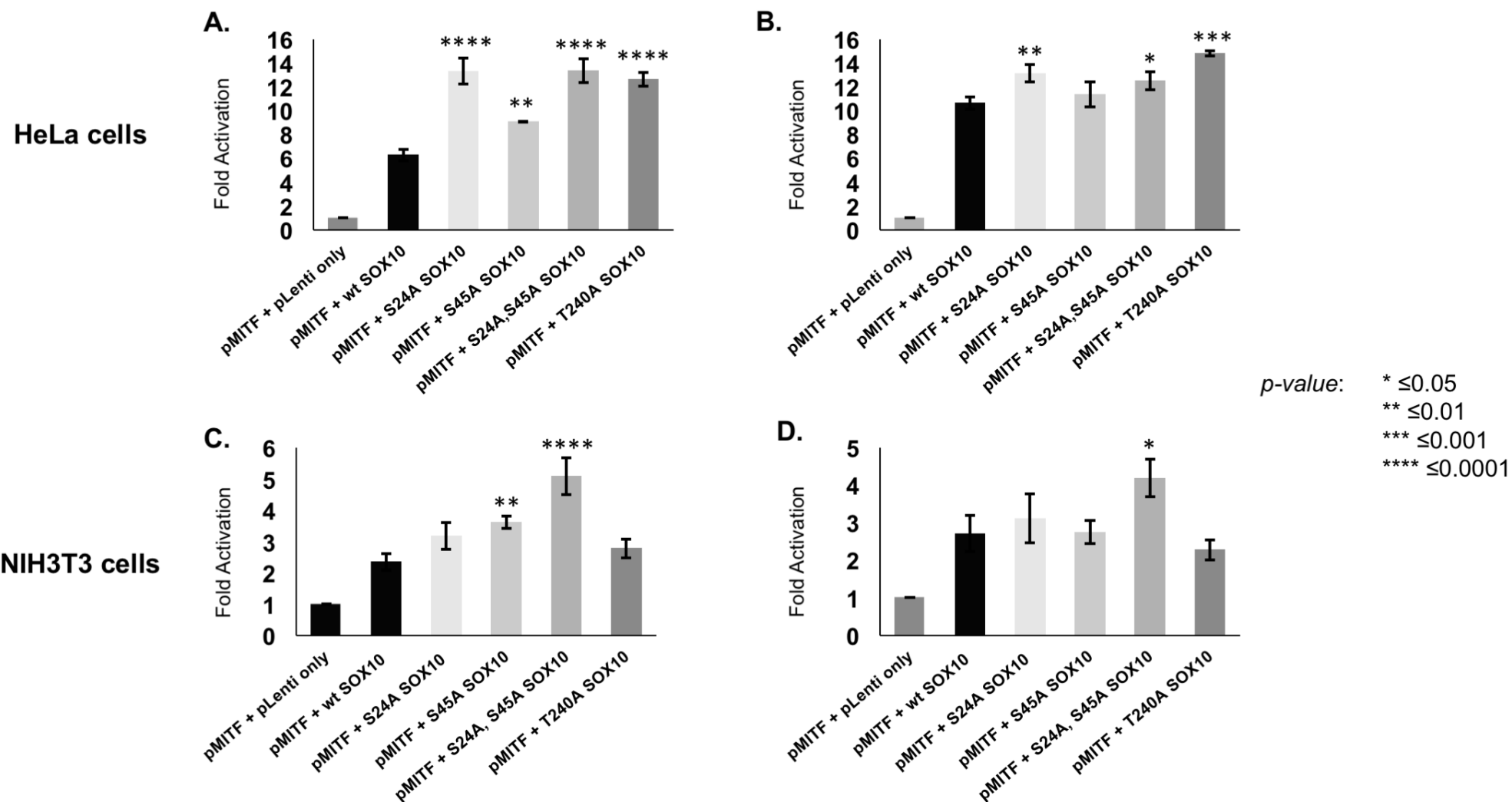

Supplement: S2 Fig — These independent replicate assays expand on data in Fig 4B and 4C. Activation of the pMITF luciferase promoter construct by the S24A and T240A SOX10 phospho-mutants was moderately but significantly increased relative to WT SOX10 in HeLa cells (top panels A and B). Activation of the pMITF construct by the S24A, S45A SOX10 double mutation construct was moderately but significantly increased relative to WT SOX10 in NIH3T3 cells (bottom panels C and D). Statistical analysis: one-way ANOVA with Bonferroni’s multiple comparison test. (PDF) [file pone.0190834.s002.pdf]

S3 Fig.

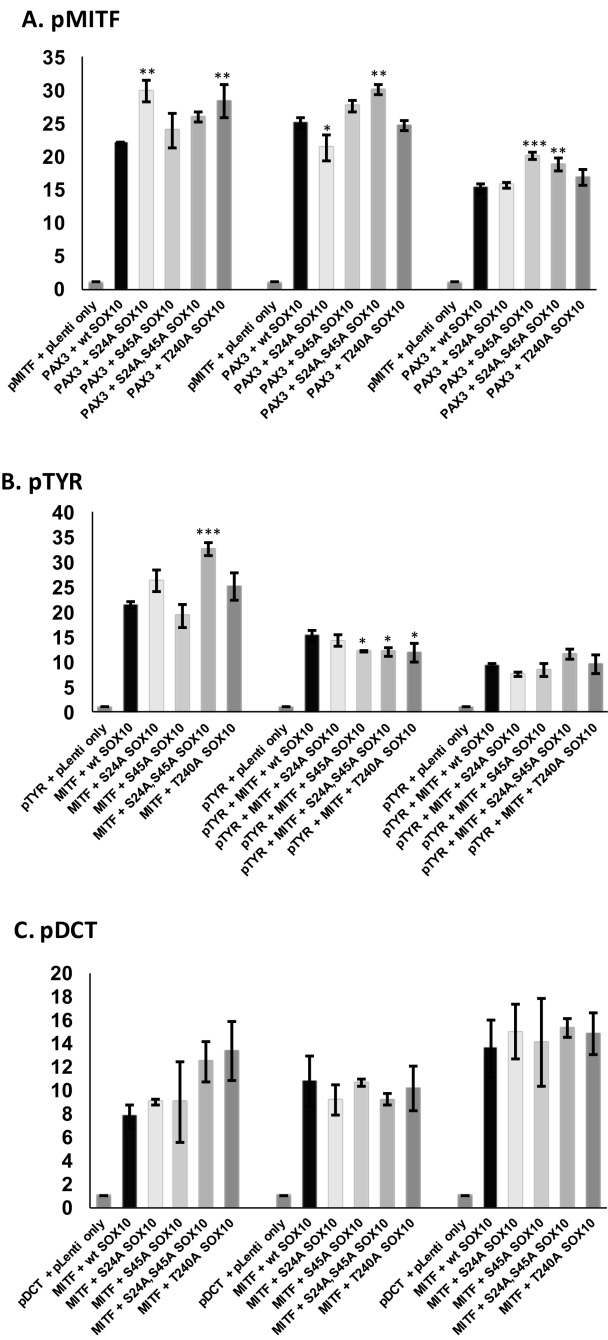

Supplement: S3 Fig — A. Synergistic activation of pMITF was achieved by all SOX10 constructs tested when co-expressed with PAX3 protein. B,C. Synergistic activation of pTYR (B) and pDCT (C) was achieved by all SOX10 phospho-mutant constructs when co-expressed with MITF. While significant differences relative to WT SOX10 were achieved in some individual samples, none of these were consistent across all biological replicates. Statistical analysis: one-way ANOVA with Bonferroni’s multiple comparison test p-value *≤0.05, **≤0.01, ***≤0.0001. (PDF) [file pone.0190834.s003.pdf]

S4 Fig: Cycloheximide pulse chase stability data for S45A and S24A, S45A SOX10 mutant proteins in 501mel cells

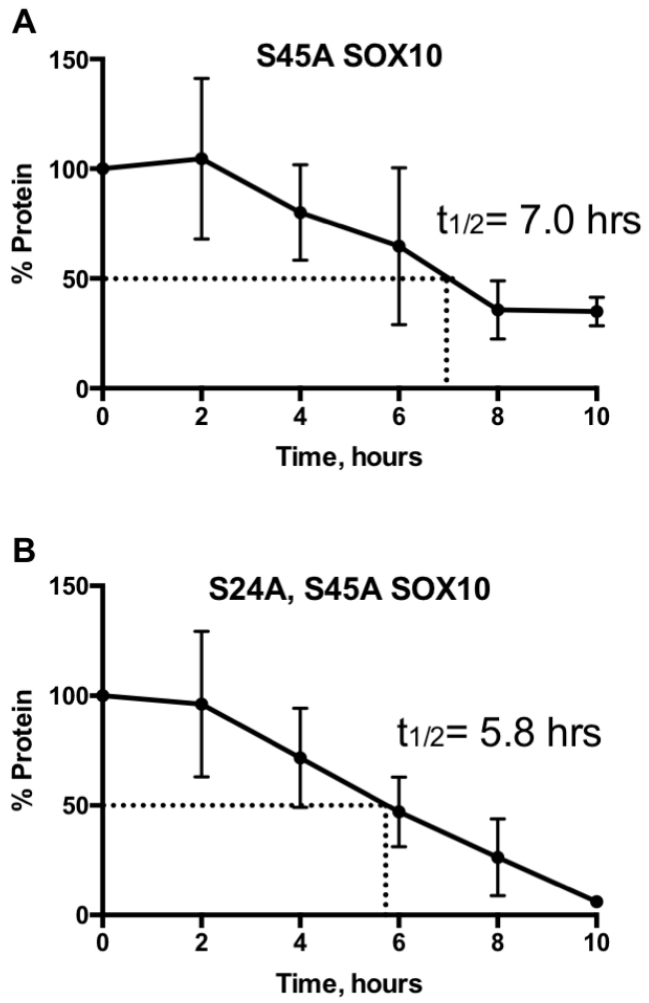

Supplement: S4 Fig — A. The S45A SOX10 phospho-mutant showed protein degradation similar to WT SOX10, with a half-life of 7 hours. B. Stability data for the S24A, S45A double mutant showed a similar half life as that of the S24A mutation alone, with a half-life of 5.8 hours. (PDF) [file pone.0190834.s004.pdf]

S5 Fig: SOXE protein phosphorylation sites cluster in similar pattern

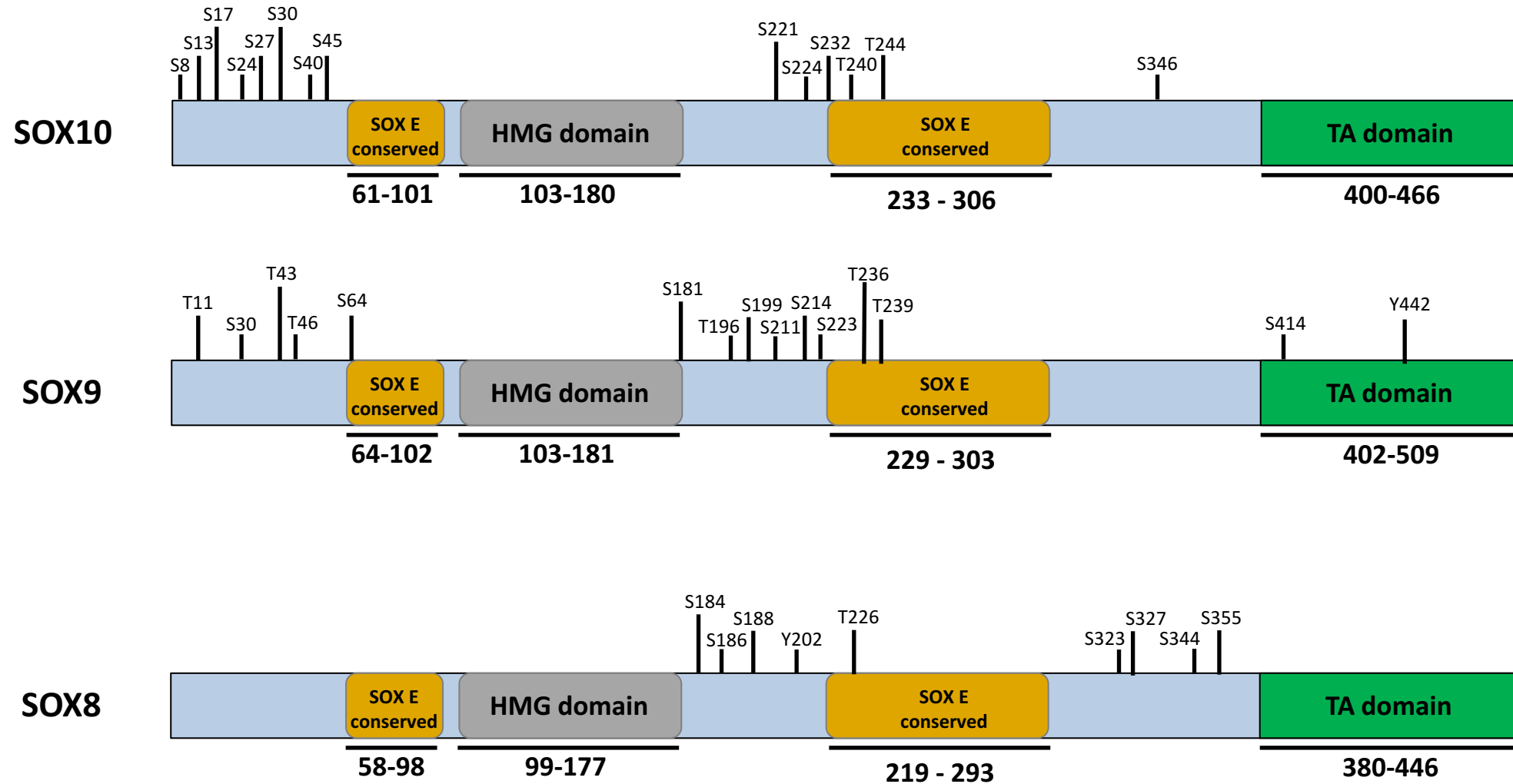

Supplement: S5 Fig — Schematic representation of all 3 SOXE proteins (SOX8, SOX9 and SOX10). Functional domains are highlighted, and phosphorylation sites have been mapped along the length of each protein (Yusuf D, Butland SL, Swanson MI, Bolotin E, Ticoll A, Cheung WA, et al. The Transcription Factor Encyclopedia. Genome Biology 2012 13:3. BioMed Central; 2012 Mar 29;13(3):R24) (PhosphoSitePlus, Cell Signaling). An N-terminal cluster of phosphorylated residues occurs proximal to the SOXE conserved dimerization region in both SOX9 and SOX10, and phosphorylation sites are clustered centrally in all 3 SOXE proteins. (PDF) [file pone.0190834.s005.pdf]

Fig 1A uncropped image

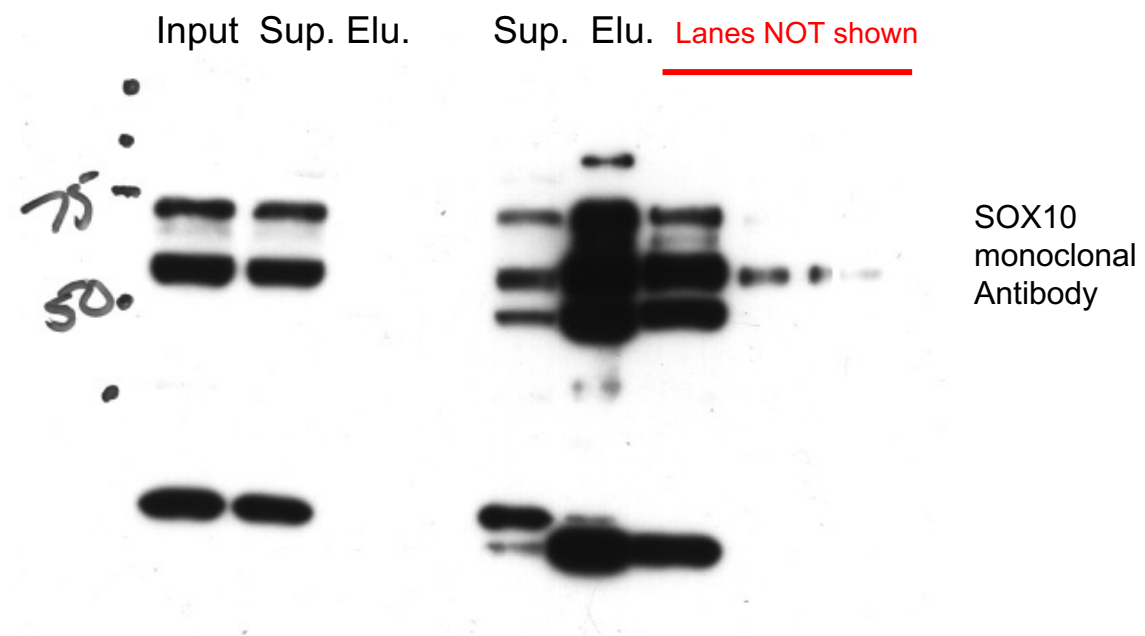

S1 Fig uncropped image

SOX10 western blot:

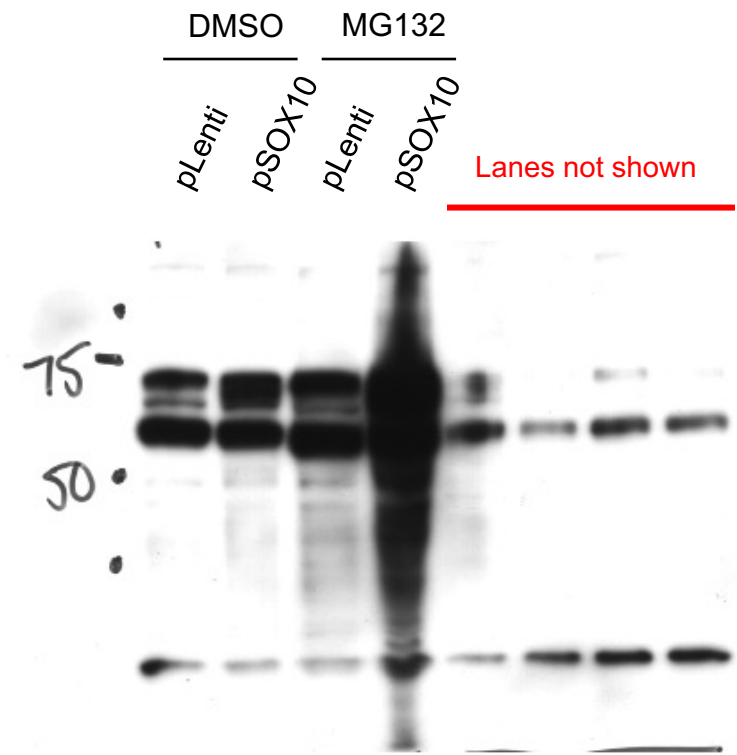

Tubulin western blot:

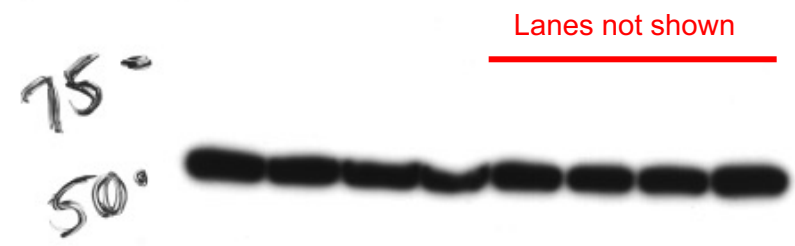

Supplement: S6 Fig — (PDF) [file pone.0190834.s006.pdf]

S1 Fig uncropped image

SOX10 western blot:

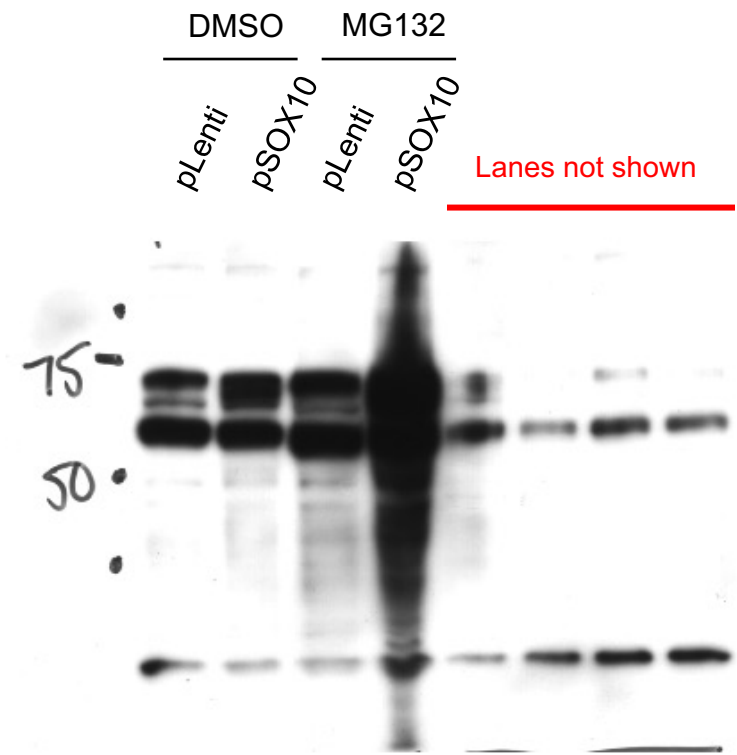

Tubulin western blot:

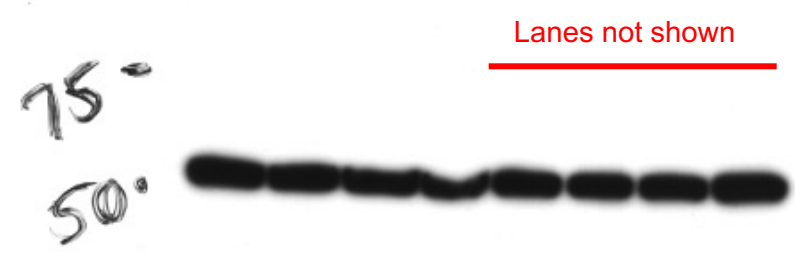

Supplement: S7 Fig — (PDF) [file pone.0190834.s007.pdf]
