# Supplementary material for: Identification and functional analysis of SOX10 phosphorylation sites in melanoma
Source: PLoS One. 2018 Jan 9;13(1):e0190834. doi: 10.1371/journal.pone.0190834 (PMC5760019; doi:10.1371/journal.pone.0190834)
Supplement: S1 Table — These include oxidation, phosphorylation and carbamidomethyl binding. Digested peptide sequences are shown, along with each modification identified within that length of amino acids. The XCorr value is the cross-correlation value from the database search; values above 2.0 typically indicate a good correlation with higher values meaning increased correlation. The DCn score is the Delta Correlation value, with numbers above 0.1 indicating good correlation. (PDF) [file pone.0190834.s008.pdf]

S1 Table: SOX10 post-translational modifications identified in Scaffold Analysis

Protein accession number	Protein identification probability	# unique peptides	# unique spectra	Sample	Peptide sequence	SEQUEST XCorr score	SEQUEST DCn score	Modifications identified by spectrum	Peptide start index	Peptide stop index
SOX10_HUMAN	100.00%	4	5	SOX10 IP_55kD band#1	ASPGPGELGK	1.85	0.532	Phospho (+80)	44	53
SOX10_HUMAN	100.00%	4	5	SOX10 IP_55kD band#1	CLSPGSAPSLGPDGGGGGSGLR	3.34	0.461	Carbamidomethyl (+57), Phospho (+80)	22	43
SOX10_HUMAN	100.00%	4	5	SOX10 IP_55kD band#1	CLSPGSAPSLGPDGGGGGSGLR	3.2	0.557	Carbamidomethyl (+57), Phospho (+80)	22	43
SOX10_HUMAN	100.00%	4	5	SOX10 IP_55kD band#1	CLSPGSAPSLGPDGGGGGSGLR	3.79	0.443	Carbamidomethyl (+57), Phospho (+80)	22	43
SOX10_HUMAN	100.00%	4	5	SOX10 IP_55kD band#1	CLSPGSAPSLGPDGGGGGSGLRASPGPGELGK	4.32	0.334	Carbamidomethyl (+57), Phospho (+80), Phospho (+80)	22	53
SOX10_HUMAN	100.00%	4	5	SOX10 IP_55kD band#1	CLSPGSAPSLGPDGGGGGSGLRASPGPGELGK	4.48	0.305	Carbamidomethyl (+57), Phospho (+80), Phospho (+80)	22	53
SOX10_HUMAN	100.00%	4	5	SOX10 IP_55kD band#1	CLSPGSAPSLGPDGGGGGSGLRASPGPGELGK	5.02	0.339	Carbamidomethyl (+57), Phospho (+80), Phospho (+80)	22	53
SOX10_HUMAN	100.00%	4	5	SOX10 IP_55kD band#1	CLSPGSAPSLGPDGGGGGSGLRASPGPGELGK	5.55	0.549	Carbamidomethyl (+57), Phospho (+80)	22	53
SOX10_HUMAN	100.00%	4	5	SOX10 IP_55kD band#1	HPGEGSPMSDGNPEHPSGQSHGPPTPPTTPK	4.27	0.292	Oxidation (+16), Phospho (+80)	216	246
SOX10_HUMAN	100.00%	4	5	SOX10 IP_55kD band#1	HPGEGSPMSDGNPEHPSGQSHGPPTPPTTPK	3.54	0.325	Oxidation (+16), Phospho (+80), Phospho (+80)	216	246
SOX10_HUMAN	100.00%	2	2	SOX10 IP_70kD band#2	CLSPGSAPSLGPDGGGGGSGLRASPGPGELGK	3.8	0.3	Carbamidomethyl (+57), Phospho (+80), Phospho (+80)	22	53
SOX10_HUMAN	100.00%	2	2	SOX10 IP_70kD band#2	HPGEGSPMSDGNPEHPSGQSHGPPTPPTTPK	3.81	0.307	Oxidation (+16), Phospho (+80)	216	246
